# Compared genomics of the strand switch region of *Leishmania *chromosome 1 reveal a novel genus-specific gene and conserved structural features and sequence motifs

**DOI:** 10.1186/1471-2164-8-57

**Published:** 2007-02-24

**Authors:** Jacques Puechberty, Christine Blaineau, Sabrina Meghamla, Lucien Crobu, Michel Pagès, Patrick Bastien

**Affiliations:** 1 CNRS/Université Montpellier I FRE 3013 "Biologie Moléculaire, Biologie Cellulaire et Biodiversité des Protozoaires Parasites", Laboratoire de Parasitologie-Mycologie, UFR Médecine, 163 Rue Auguste Broussonet, 34090 Montpellier, France; 2 Service de Génétique Médicale, Centre Hospitalier Universitaire, Montpellier, France

## Abstract

**Background:**

Trypanosomatids exhibit a unique gene organization into large directional gene clusters (DGCs) in opposite directions. The transcription "strand switch region" (SSR) separating the two large DGCs that constitute chromosome 1 of *Leishmania major *has been the subject of several studies and speculations. Thus, it has been suspected of being the single replication origin of the chromosome, the transcription initiation site for both DGCs or even a centromere. Here, we have used an inter-species compared genomics approach on this locus in order to try to identify conserved features or motifs indicative of a putative function.

**Results:**

We isolated, and compared the structure and nucleotide sequence of, this SSR in 15 widely divergent species of *Leishmania *and *Sauroleishmania*. As regards its intrachromosomal position, size and AT content, the general structure of this SSR appears extremely stable among species, which is another demonstration of the remarkable structural stability of these genomes at the evolutionary level. Sequence alignments showed several interesting features. Overall, only 30% of nucleotide positions were conserved in the SSR among the 15 species, versus 74% and 62% in the 5' parts of the adjacent *XPP *and *PAXP *genes, respectively. However, nucleotide divergences were not distributed homogeneously along this sequence. Thus, a central fragment of approximately 440 bp exhibited 54% of identity among the 15 species. This fragment actually represents a new *Leishmania*-specific CDS of unknown function which had been overlooked since the annotation of this chromosome. The encoded protein comprises two trans-membrane domains and is classified in the "structural protein" GO category. We cloned this novel gene and expressed it as a recombinant green fluorescent protein-fused version, which showed its localisation to the endoplasmic reticulum. The whole of these data shorten the actual SSR to an 887-bp segment as compared with the original 1.6 kb. In the rest of the SSR, the percentage of identity was much lower, around 22%. Interestingly, the 72-bp fragment where the putatively single transcription initiation site of chromosome 1 was identified is located in a low-conservation portion of the SSR and is itself highly polymorphic amongst species. Nevertheless, it is highly C-rich and presents a unique poly(C) tract in the same position in all species.

**Conclusion:**

This inter-specific comparative study, the first of its kind, (a) allowed to reveal a novel genus-specific gene and (b) identified a conserved poly(C) tract in the otherwise highly polymorphic region containing the putative transcription initiation site. This allows hypothesising an intervention of poly(C)-binding proteins known elsewhere to be involved in transcriptional control.

## Background

The protozoan parasite *Leishmania sp*. is responsible for significant worldwide human morbidity and mortality, and the clinical features of diseases vary depending on the causative species. The genus *Leishmania *belongs to the family of trypanosomatids with, among others, *Trypanosoma brucei *and *T. cruzi*, responsible for sleeping sickness and Chagas' disease, respectively. Taxonomically, it is separated into two sub-genera:*L*. (*Leishmania*) and *L. (Viannia*). The differentiation between both appears very ancient, as it is estimated to be contemporary to the formation of the Gondwana, i.e. around 60 million years [1]. However, comparative genomics showed the general structure of the genome to be remarkably stable within this genus. Chromosomal synteny groups are entirely conserved for all Old World species (subgenus *L*. (*Leishmania*)) where 36 heterologous chromosomes have been identified [2]. As regards the New World, only two and one large chromosomal rearrangements, according to the subgenus, have been shown as compared with Old World species. This leads to a chromosome number of 34 and 35 for *L*. (*Leishmania*) and *L. (Viannia*) species, respectively, but with all Old World species linkage groups remaining conserved [3]. Similarly, chromosomal restriction maps showed a complete conservation of collinearity of markers between species [4]. Finally, the sequencing of the 'TriTryp' genomes also demonstrated a high degree of synteny among those three protozoa (*L. major, T. brucei *and *T. cruzi*) [5].

Trypanosomatids exhibit a number of highly original molecular and cellular biological features. Among those, one may cite systematic trans-splicing, consisting in the addition of a 39 nt-spliced leader RNA at the 5'end of all mRNAs, and the near absence of promoters for polymerase II, implying an absence of regulation of gene expression at the transcriptional level. One of the most extraordinary features revealed by these genome projects was the gene organisation into large collinear clusters present on a single strand and comparable to prokaryotic polycistronic units, except that the genes present have no common nor akin function [6-8]. These large directional gene clusters (DGCs) are separated by short sequences of a few kbs termed coding strand switches or strand-switch regions (SSRs), where the transcription sense converges or diverges. This remarkable position led several authors to express different hypotheses upon the putative function of these regions in *Leishmania*.

The comparative *in silico *analysis of several SSRs in *L. major *only revealed low homologies at the structural as well as nucleotide sequence level [5,8,9] that render difficult a common interpretation as for their putative role. AT distribution and hairpin content analysis failed revealing common features to different SSRs but allowed separating them into two groups with respect to the adjacent gene clusters transcription orientation (divergent or convergent) [9]. The same authors showed that the five SSRs analysed presented a very high intrinsic DNA curvature, the latter being classically associated with transcription as well as replication and centromere functions (reviewed in [7]).

However, experimental data showed that the deletion of the sole SSR of chromosome 1 did not affect mitotic stability, hence it was not necessary for chromosomal replication nor segregation [10]. This goes against the hypotheses of this SSR being a single replication origin [11] or a centromere [9]. It is noteworthy that, whereas in *T. cruzi*, a 16-kb SSR, made for a large part of retroelements, has been identified as a centromere [12], in *Leishmania*, the centromeric function on chromosome 1 could be attributed to a subtelomeric 20-kb satellite repeat cluster [13]. On the other hand, run-on experiments showed that this same SSR on *L*. major chromosome 1 contained a single site of bi-directional initiation of transcription for both gene clusters [14]. This might explain why the deletion of this SSR could not be realised on the three copies of chromosome 1 in the reference strain *L. major *'Friedlin' [10]. On the other hand, the expression of a reporter gene inserted into one of both gene clusters was not affected by the deletion of the SSR [10], which shows that transcription is possible without the presence of the SSR. Myler et al. explained the latter fact by a minor 'residual' level of expression on the chromosome that would not initiate at the SSR itself [14]: still, this level of transcription is sufficiently high to allow the expression of the reporter protein.

Here, we bring new elements in the knowledge of the structure and conservation of these regions using an infrageneric compared genomics approach. We have sequenced the SSR of chromosome 1 in 15 highly divergent species of *Leishmania *and *Sauroleishmania *(of which the inclusion within *Leishmania *remains uncertain) [15] and show the presence of conserved structural elements and motifs that were overlooked during sequence annotation or using inter-genera comparative genomics.

## Results and discussion

### The general structure of the "switch region" is conserved in all Leishmania species

**Figure 1:**
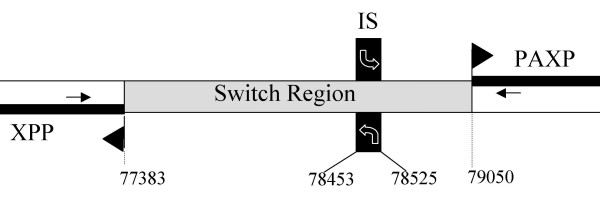
**Schematic representation of the strand switch region (SSR) of chromosome 1 in *L. major *Friedlin**. The nt positions (figures) refer to the complete sequence of this chromosome available on the GeneDB website [28]. *XPP*: acidocalcisomal exopolyphosphatase gene; *PAXP*: poly(A) export protein gene; IS: putative transcription initiation site, with the curved arrow showing transcription sense [14]. The start codons of both genes are indicated by a closed arrowhead. It is noteworthy that, after careful compared analysis with the genomes of *Trypanosoma brucei *and *T. cruzi*, we repositioned the start codon of *PAXP *at position 79049 and not at 79145 as wrongly indicated on GeneDB. The positions of the primers on the *XPP *and *PAXP *genes are indicated as arrows.

We have analyzed the SSR of chromosome 1 in 15 species of *Leishmania *and *Sauroleishmania*. In each species, this region was amplified by using PCR primers located on the 5' part of the two genes adjacent to the SSR in *L. major*: *PAXP *and *XPP *(Fig. 1). For all species, a fragment of expected size (approximately 1650 bp) was found. This fragment was cloned and sequenced [GenBank™ accession numbers DQ522034 to DQ522048]: this confirmed the presence of both genes adjacent to the SSR. As expected, the transcription direction of both genes is in opposite sense and directed towards the telomeres in all species. The distance between the start codons of both genes is relatively constant as it lies between 1640 and 1669 bp (for *L. aethiopica *and *L. amazonensis *respectively) (Table 1). Incidentally, our data allow repositoning the start codon of the *PAXP *gene at position 79049 instead of 79145. The SSR is thus remarkably size-stable since it varies of <2% among both extremes. These results support previous studies on the conservation of chromosomal linkage groups [2,3], or the compared mapping of certain chromosomes in various species [4], showing a surprising interspecific conservation of the organization of these genomes. As described in *L. major *[6], the AT content of this sequence in the various species is relatively high since it ranges between 50% (*L. enriettii*) and 54% (*Sauroleishmania*) (Table 1). This relative AT-richness distinguishes the SSR from the remainder of the genome where it is 35% [6]. This physical characteristic may facilitate weaker binding of the two strands during either transcription or replication processes, and is classically found in the non-coding regions with a structural role such as centromeres or replication origins [16,17]. The whole of these features shows that the general structure of this region is highly conserved among the 15 species studied here.

### Presence of a conserved CDS in the switch region

**Figure 2:**
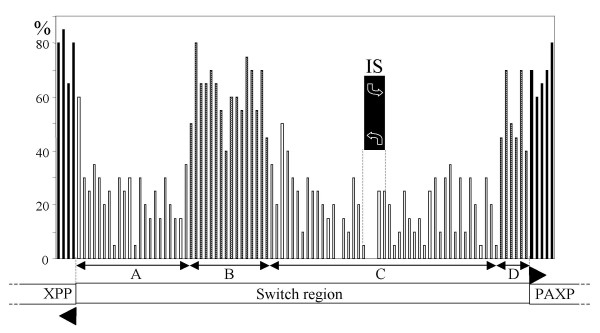
**Histogram representation of the rates of identical nt positions amongst the SSR sequences of 15 *Leishmania *species**. Each vertical bar represents a 20 nt window. At each end, 109 and 97 bp cover the 5' ends of *PAXP *and *XPP *genes, respectively (black bars). Two segments of the SSR appear highly conserved (B and D, hatched bars) as compared with other segments (A and C, open bars). It is noteworthy that the comparison of 100 bps from a *Leishmania *gene generally yields divergence rates similar to those obtained when one compares the whole length of the gene : e.g. here, for *PAXP*, the identity rates between the sequences from *L. major *and *L. braziliensis *are 83.5% for the whole gene (now available in GeneDB) and 85.7% for the 109 bps analysed here. XPP, PAXP, IS as in Fig. 1.

An alignment of the 15 sequences amplified as above was realised using ClustalW software. Figure 2 represents the rates of conserved nucleotides (nt) among the 15 species studied, at the level of the SSR on the one hand, and of the adjacent genes on the other hand. The 5' part of both genes is highly conserved, with a mean identity rate among all 15 species of 74% and 62% for *XPP *and *PAXP *respectively (analysed on 97 and 109 bp, respectively); most mutations being silent, this yields identity rates at the amino acid level of 80 % and 78 % respectively. It should be noted that the comparison of 100 bps from a *Leishmania *gene generally yields divergence rates representative of those obtained when one compares the whole length of the gene (see Legend of Fig. 2). At the level of the SSR, the overall nt divergence appears much more significant: only 30% of nt positions are conserved among the 15 sequences. Interestingly, this divergence is not distributed homogeneously along this sequence. Thus, a central fragment of approximately 440 bp (429 bp in *L. major*), termed B, exhibits 54% of identity among the 15 species (Table 1 and Fig. 2). It contrasts with most of the rest of the SSR (except fragment D, see below) where this percentage is much lower, being 23% and 22% for fragment A (left) and C (right), respectively. The quality of the alignment of fragment B is actually close to that observed in the 5' parts of the *XPP *and *PAXP *genes (Fig. 2). A closer analysis revealed that it actually corresponds to an ORF conserved in the whole of the species, hence to a CDS [see Additional file 1: Amino acid sequence alignment], whose transcription orientation would be towards the 'left' of the chromosome. BLAST analysis did not show any valid homology of this sequence to any other SSR not to any other organism, in particular in the genomes of *T. brucei *and *T. cruzi*. This CDS, with no putative function, was recently added in the *L. major *GeneDB database.

Another moderately conserved fragment, termed D, was also noted in the ca. 120 nucleotides upstream of the *PAXP *gene (Fig. 2) in the SSR. Of note on this fragment is the putative spliced leader RNA addition site (AG) for *PAXP *[14] which is conserved in all 15 species. By contrast, it is noteworthy that the putative trans-splicing acceptor site (SAS) of the *XPP *gene, that was identified in the same report 840 bp upstream of the methionine codon [14], is not conserved amongst all species (only in 9/15 species) and is now located 50 nt upstream of the newly identified CDS, hence was probably misidentified. The putative conserved SAS of the new CDS is likely located 20 nt upstream of its start codon. The sequence alignment allows the identification of only one candidate SAS for the *XPP *gene that would be conserved amongst all *Leishmania *species (but not *Sauroleishmania*), 50 nt upstream (*L. major *sequence) of the start codon of *XPP*.

### Analysis of the *Leishmania*-specific CDS

As it is is not conserved among the Tri-Tryps (*L. major*, *T. brucei *and *T. cruzi*), this CDS therefore appears *Leishmania*-specific. As such, it constitutes one example of a species-specific gene occurring at a synteny breakpoint between these three organisms, since the SSR analysed here forms such a breakpoint [5]. In *T. cruzi*, this SSR is conserved as such (1211 bp, with the *XPP *and *PAXP *gene located on the 'left' and 'right' of the SSR respectively); but another unknown CDS is located upstream of the PAXP gene, hence at the start of the 'right-oriented' directional gene cluster (DGC) (like our novel gene is located at the start of the 'left-oriented' DGC in *Leishmania*). This CDS in *T. cruzi *is conserved in the same location and DGC in *T. brucei*, suggesting that our novel gene likely is the result of both a gene deletion (that of the CDS of *T. cruzi *and *T. brucei*) and a gene insertion (that of our novel CDS) events during evolution. By contrast, the region is not conserved as such in *T. brucei*, where it sizes 8286 bp, bears several 'unlikely' CDSs and retroelements and, more importantly, does not actually constitute a SSR; still, it is flanked downstream by the same DGC as in the other Tri-Tryps and upstream by the upstream DGC of *L. major *chromosome 1 but entirely inverted [5].

The function of this new gene is unknown (like>60 % of the *Leishmania *genes). The Protfun program allowed predicting its putative function in either energy metabolism or cell enveloppe, and classified it in the "structural protein" GO category. Further bioinformatic analysis showed that the encoded 143 amino acid protein (in *L. major*) comprises two transmembrane helices (residues 32–54 and 104–126; probablity = 0.99 using TMHMM and InterProScan software). As regards post-transcriptional processing, a signal peptide was identified at the N-terminus (according to Signal-P); and two serine and one threonine are potential phosphorylation sites (according to NetPhos). Finally, TargetP shows a strong prediction for a mitochondrial localisation, but targeting signals are often non-conserved in Trypanosomatids [[18]; our unpublished data].

**Figure 3:**
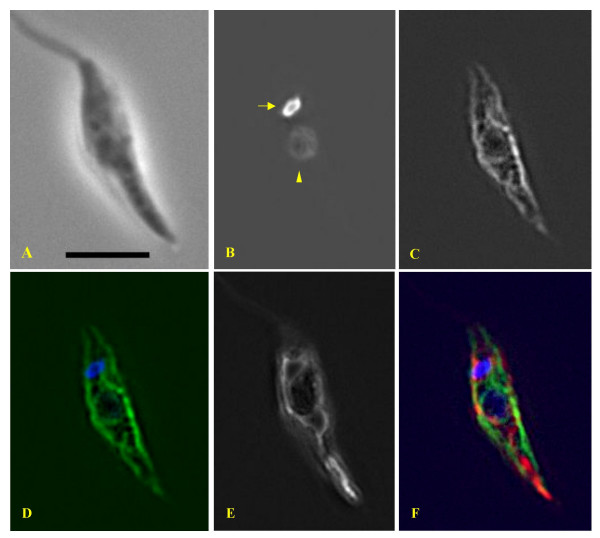
**The protein encoded by the novel *Leishmania*-specific gene localises to the endoplasmic reticulum**. Images of an *L. major *'Friedlin' promastigote expressing a recombinant GFP-fused version of the protein encoded by the novel CDS. (A) Phase contrast microscopy; scale bar : 10 microns. (B) DAPI-staining of the nucleus (arrowhead) and the single mitochondrial DNA or kinetoplast (arrow). (C) Localisation of the GFP-fused protein viewed in fluorescence. (D) Colour combination of GFP (green) and DAPI (blue) fluorescence. (E) Mitochondrion labeled with Mitotracker™. (F) Merged picture: blue = DNA; red = Mitotracker™; green = GFP-fused protein. The protein localised to a subpellicular, cytoplasmic and perinuclear network that is clearly different from the mitochondrion and closely resembles the endoplasmic reticulum; the latter being identified by expression of the plasmid construct GFP-MDDL that acts as an endoplasmic reticulum retention signal in trypanosomatids [31] [see Additional File 2].

We then constructed an episomal vector expressing a recombinant green fluorescent protein (GFP)-fused version of the protein after transfection in *L. major*, in order to observe its subcellular localisation. Combined cell staining wih Mitotracker showed that, in opposition with the predictions of TargetP, the protein is not addressed to the mitochondrion (Fig. 3). By contrast, it localises to a subpellicular, cytoplasmic and perinuclear network, that is clearly not overlapping the mitochondrion, most likely the endoplasmic reticulum. This localisation is compatible with the presence of two transmembrane domains. It is noteworthy that no phenotype (cell growth, cell cycle, morphology) could be associated with the episomal overexpression of the protein (not shown).

### The putative transcription initiation site is located in a highly variable segment

**Figure 4:**
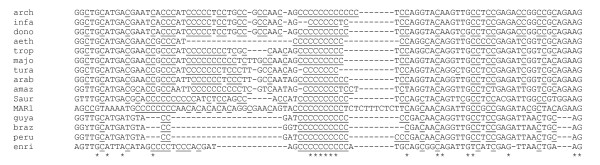
**Alignment of the sequences corresponding to the putative bidirectional transcription initiation site among the 15 *Leishmania *species analysed**. Alignment of the sequences corresponding to the putative bidirectional transcription initiation site [14] among the 15 *Leishmania *species analysed. This shows the presence of a unique and conserved poly(C) in all species studied here. Cytidines are shown underlined. Asterisks indicate the nucleotides conserved in all species. The first four letters of each line indicate the species name (see Table 1).

Martinez-Calvillo et al. [14] identified a 73-bp segment on the SSR as the putative transcription initiation site on chromosome 1 (position 78453–78525). Fig. 4 presents the inter-specific alignment of the nt sequences corresponding to this segment. Interestingly enough, this sequence is located in the portion of the SSR with the highest inter-specific nt divergence (IS in Fig. 2). Moreover, the sequence itself is highly polymorphic amongst the various species (Fig. 4). However, this segment shows notable conserved features in all species: (i) as noted previously in *L. major *[14], it has the highest GC rate (ca. 70%) in the whole SSR, and particularly is highly C-rich; (ii) it presents a unique poly(C) tract conserved in the same position, although of variable length, in all species, including in highly divergent species like *L. enriettii *or MAR1 (see below). The presence of this poly(C) tract was not specifically mentioned in the corresponding paper [14]. Although it may be considered as a simple polypyrimidine tract, it is the only conserved tract in the whole segment, and this conserved feature makes it is tempting to speculate upon its possible role. The high C-content may here induce a conformation of the DNA double helix supporting RNA polymerase entry. Moreover, poly(C)-binding proteins, a conserved subfamily of K-homology domain-containing proteins, are known, among other functions, to be involved in transcriptional control through a variety of mechanisms [19] and in particular to activate transcription of the human *c-myc *gene [20].

### The switch region sequences alignment yields a coherent taxonomic tree

**Figure 5:**
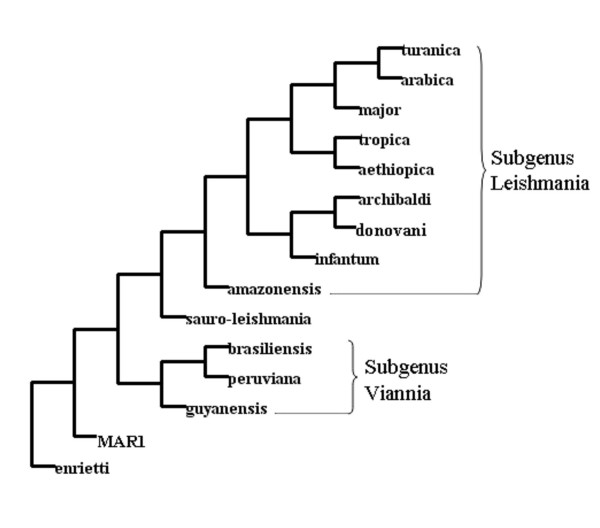
**Phylogenetic analysis of 15 divergent *Leishmania *species obtained from the chromosome 1 strand switch region sequence alignment**. Unrooted tree produced from the chromosome 1 strand switch region sequence alignment by the DNAMP program in DAMBE (see text for comments). Names represent different *Leishmania *species (see Table 1).

The phylogenetic tree built from these SSR sequences clearly identified the various groups classically defined in the genus *Leishmania *(Fig. 5) [15]. Thus, a very high level of homology can be observed within groups that comprise classically very closely related species such as *L. major-L. arabica-L. turanica *and *L. peruviana-L. brasiliensis-L. guyanensis*, with 98.8% and 97.6% of identical nucleotide positions over the whole SSR, respectively. Between these two groups, which may be taken as representatives of the *L. (Leishmania*) and *L. (Viannia*) subgenera respectively, the percentage of identity is only 69%, supporting the current taxonomic dichotomy. A homology rate of 83.7 % for this SSR had previously been reported among *L. major*, *L. donovani*, *L. infantum*, *L. mexicana *and *L. amazonensis *[14]. Interestingly, two species are shown clearly differentiated at the basis of the tree: *L. sp*. MAR1 and *L. enriettii*. The first one corresponds to extremely rare isolates from cutaneous leishmaniasis patients from the Caribbean [21]. The second one is an animal species isolated on rare occasions from the guinea-pig [22] and was found as the most external member of the genus *Leishmania *[23]. Both species also clustered together at a basal position for all other Euleishmania by molecular phylogeny using DNA polymerase alpha and RNA polymerase II gene-encoding sequences [15]. Finally, it is noteworthy that these sequence alignments strongly support the inclusion of *Sauroleishmania *(of which the taxonomic position remains controversial; reviewed in [1]) within *Leishmania *and perhaps in an intermediary position between both subgenera.

## Conclusion

This study is the first of its kind in Trypanosomatids as it is based on an 'inter-specific' study (comparing 15 *Leishmania *species), as opposed to the vast analysis that had been published in 2005 that compared the "Tri-Tryps", *L. major, T. brucei *and *T. cruzi *[5]. The interest of the first approach is to identify *Leishmania*-specific genes that would have been overlooked in the second approach, e.g. this new CDS. This is a novel demonstration of the interest of a (here infrageneric) compared genomics approach in identifying unknown genes or functional motifs. The presence of this CDS might partly explain the difficulties encountered in knocking-out the SSR [10], if this endoplasmic reticulum-restricted *Leishmania*-specific gene had an essential role.

Considering its transcription sense, these data also actually shortens the 'proper' strand switch region to an 877-bp segment (segment C in Fig. 2, position 78173 to 79049 on *L. major *Friedlin chromosome 1).

This study also sheds new light upon the putative function of the SSR of chromosome 1. Surprisingly indeed, the putative transcription initiation site (TIS) previously identified on this chromosome by run-on analysis [14] is found in the most polymorphic portion of the SSR; yet, it presents a conserved poly(C) tract in a highly conserved position in divergent *Leishmania *species. These data both question and reinforce the TIS hypothesis. The high nt sequence polymorphism of this segment and the ubiquitous presence of poly(C) tracts make it difficult to define structural features of TISs in *Leishmania*. Conversely, the fact that a number of genes encoding K-homology domain-containing proteins have been identified within the *Leishmania *genome make it possible to hypothesize that these proteins participate in transcriptional control. Experimental analysis of these genes may help in understanding transcription initation mechanisms used by this unusual parasite.

## Methods

### Parasite strains

The *Leishmania *strains used for sequencing the switch region of chromosome 1 were: in the sub-genus *L. (Leishmania*), *L. donovani *LEM138 (MHOM/IN/00/DEVI), *L. infantum *LEM1136 (MHOM/FR/87/LEM1136-cl), *L. archibaldi *LEM1005 (MHOM/ET/72/GEBRE1), *L. major *LEM134 (MHOM/SU/73/5-ASKH), *L. major *'Friedlin' (MHOM/IL/81/FRIEDLIN), *L. turanica *LEM558 (MRHO/SU/74/95A), *L. arabica *LEM1108(MPSA/SA/83/JISH220), *L. aethiopica *LEM1660 (MHOM/ET/89/LEM1660-CL), *L. tropica *LEM84 (MRAT/IQ/72/ADHANIS1), *L. amazonensis *LEM2246 (IFLA/TT/71/71-110), *L. enriettii *LEM1120 (MCAV/BR/45/L88) and *L. sp*. MAR1 or LEM2494 (MHOM/MQ/92/MAR1) [22]; in the sub-genus *L. (Viannia*), *L. braziliensis *LEM396 (MHOM/BR/75/M2903), *L. peruviana *LEM1535 (MHOM/PE/84/UN59) and *L. guyanensis *LEM85 (MHOM/GF/79/LEM85); and in the genus *Sauroleishmania, S. tarentolae *LEM351 (RTAR/DZ/39/TAR-VI). All strains were cultivated on blood agar (Novy-McNeal-Nicolle) medium. Their identity was checked by examination of 15 isoenzyme systems immediately prior to this study [24].

### PCR conditions and sequences

Genomic DNA extraction of each strain was performed by phenol-chloroform, followed by ethanol precipitation. All DNA regions analysed were PCR-amplified from total genomic DNA using the high -fidelity Platinum^® ^Pfx DNA Polymerase (Invitrogen^®^). Different primers were used for targeting different areas of the acidocalcisomal exopolyphosphatase (*XPP*) gene, the poly(A) export protein (*PAXP*) gene and the central part of the SSR: forward primers ccgacaatgctgtccatgt and gtggcaatgcaaatgggcagc; and reverse primers gcaactcccgtcccacga, and tgagcgcgcgacttgtcg. After electrophoresis, PCR products were purified from agarose gels and cloned into pGEMT-Easy (Promega^®^), then sequenced on a Licor^® ^automated sequencer and later assembled using the Sequencher™ software (Gene Codes Corp.^®^). All sequences were double strand reads and the quality of the sequence obtained was carefully checked manually.

### Sequence analysis

Nucleotide sequence alignments and phylogenetic trees were realised using ClustalW [25] and Dambe software [26]. Homologies were searched via Blast on two distinct websites [27,28]. Bioinformatic analysis of the protein was done using CBS [29] and EBI [30] software resources.

### Expression of the recombinant protein

The coding region of the novel CDS, with the start and stop codons removed, was PCR-amplified from *L*.*major *'Friedlin' genomic DNA with specific forward and reverse oligonucleotides containing the MfeI and HpaI restriction sites respectively. The PCR product, purified and digested with MfeI-HpaI, was cloned into the MfeI and HpaI sites of the plasmid vector pTH_6_nGFPc [18], generating a construct where the CDS is fused to the *GFP *gene in its 3' end. 100 μg of episomal DNA plasmid were then transfected by electroporation into 8 × 10^7 ^*L. major *'Friedlin' promastigotes grown to mid-log phase, which were grown under selection pressure with hygromycin at 30 μg/ml. *Leishmania *cells were then viewed in microscopy and photographed as described [18]. The mitochondrion was visualised by incubating cultures in MitoTracker Red CMXRos (Molecular Probes^®^) for 10 min prior to fixation.

## Authors' contributions

JP and SM carried out the molecular genetic studies and participated in the sequence alignment. CB participated in the sequence alignment and analysis. CB and LC constructed the plasmid vectors and performed the transfection analysis. SM and MP drafted the manuscript. MP participated in the design of the study and performed the sequence data analysis. PB conceived the study, and participated in its design and coordination and helped to draft and finalize the manuscript. All authors read and approved the final manuscript.

## Supplementary Material

Additional File 1 Alignment of the amino acid sequences of the novel CDS among 15 *Leishmania *species. The data provided represent the alignment of the amino acid sequences of the novel CDS identified in the central part of the chromosome 1 switch region among 15 *Leishmania *species. Colour codes indicate groups of amino acids (small, acidic, basic...) and are explicited on the EBI website [32]. "*" residues identical in all sequences; ":" conserved substitutions; "." semi-conserved substitutions. Species are indicated by their three first letters (see Table 1), except *L. sp*. MAR1 shown as "anc" (standing for "ancestral"). Click here for file

Additional File 2 Visualisation of the endoplasmic reticulum (ER) in *Leishmania major *using an ER-marker. The pictures provided show an *L. major *cell where the ER was visualised using an ER-specific expression plasmid construct. Images of an *L. major *promastigote form expressing the plasmid construct GFP-MDDL that acts as an endoplasmic reticulum retention signal in trypanosomatids [31]. Upper left: phase contrast microscopy; scale bar : 10 microns. Upper right (DAPI): DAPI-staining of the nucleus and kinetoplast. Lower left (BIP-GFP): Localisation of the GFP-MDDL marker viewed in fluorescence. Lower right (MERGE): Colour combination of GFP (green) and DAPI (red) fluorescence. Click here for file

## Figures and Tables

**Table 1:** Comparative features of the chromosome 1 strand switch region and the 5'-ends of the adjacent *XPP *and *PAXP *genes in 15 *Leishmania *species ^a^.

**Species**	**XPP**		**Switch region**			**PAXP**	
	% nt div. ^b^	div. aa/32aa ^c^	Size (bp)	% AT	% nt div ^d^	% nt div. ^e^	div. aa/36aa ^f^

*L. aethiopica*	1	1	1640	51	15	1	0
*L. arabica*	1	0	1658	50	16	4	1
*L. turanica*	2	1	1665	50	22	2	0
*L. major*	3	0	1666	50	16	5	1
*L. tropica*	2	0	1659	50	14	2	1
*L. archibaldi*	1	0	1668	51	16	3	1
*L. donovani*	1	0	1666	50	14	2	1
*L. infantum*	1	0	1662	50	15	3	1
*L. amazonensis*	3	0	1669	52	16	2	0
*Sauroleishmania*	9	3	1643	54	25	4	0
*L. peruviania*	3	1	1656	53	23	13	4
*L. braziliensis*	3	1	1657	53	24	10	3
*L. guyanensis*	3	1	1652	53	23	11	3
*L. sp. MAR1*	12	4	1658	51	30	12	0
*L. enriettii*	6	1	1642	50	25	8	0

^a ^For each species, the divergent nucleotides or amino acids were counted as compared with the consensus sequence; ^b ^Percentage of nucleotide (nt) divergence established from the sequence of 97 nt at the 5' end of the gene; all percentages were rounded up to the closest integer number; ^c ^Number of unconserved amino acids (aa) out of 32 aa at the N-terminal end of the protein; ^d ^Percentage of nucleotide (nt) divergence established from the consensus sequence over the whole SSR as defined in the Material and Methods; ^e ^Percentage of nucleotide (nt) divergence established from the sequence of 109 nt at the 5' end of the gene; ^f ^Number of unconserved amino acids (aa) out of 36 aa at the N-terminal end of the protein.
