# Supplementary figures and images for: Compared genomics of the strand switch region of Leishmania chromosome 1 reveal a novel genus-specific gene and conserved structural features and sequence motifs
Source: BMC Genomics. 2007 Feb 24;8:57. doi: 10.1186/1471-2164-8-57 (PMC1805754; doi:10.1186/1471-2164-8-57)

## Slide 1
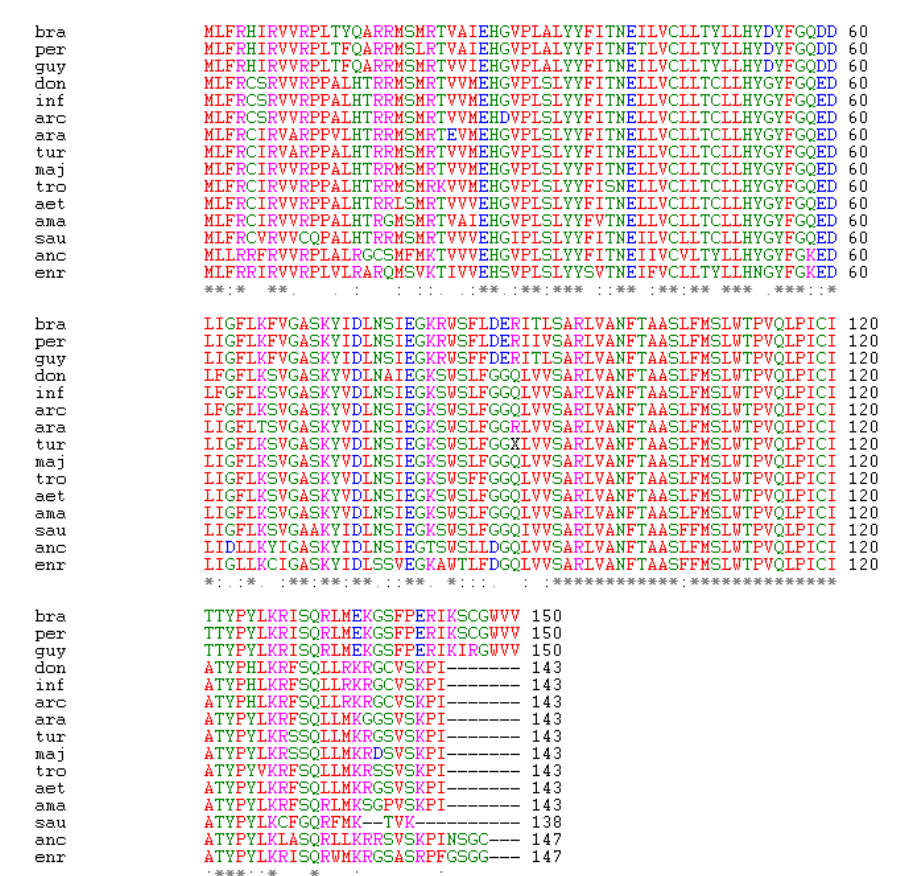

Supplement: Additional File 1 — Alignment of the amino acid sequences of the novel CDS among 15 Leishmania species. The data provided represent the alignment of the amino acid sequences of the novel CDS identified in the central part of the chromosome 1 switch region among 15 Leishmania species. Colour codes indicate groups of amino acids (small, acidic, basic...) and are explicited on the EBI website [32]. "*" residues identical in all sequences; ":" conserved substitutions; "." semi-conserved substitutions. Species are indicated by their three first letters (see Table 1), except L. sp. MAR1 shown as "anc" (standing for "ancestral"). [file 1471-2164-8-57-S1.ppt]

## Slide 1
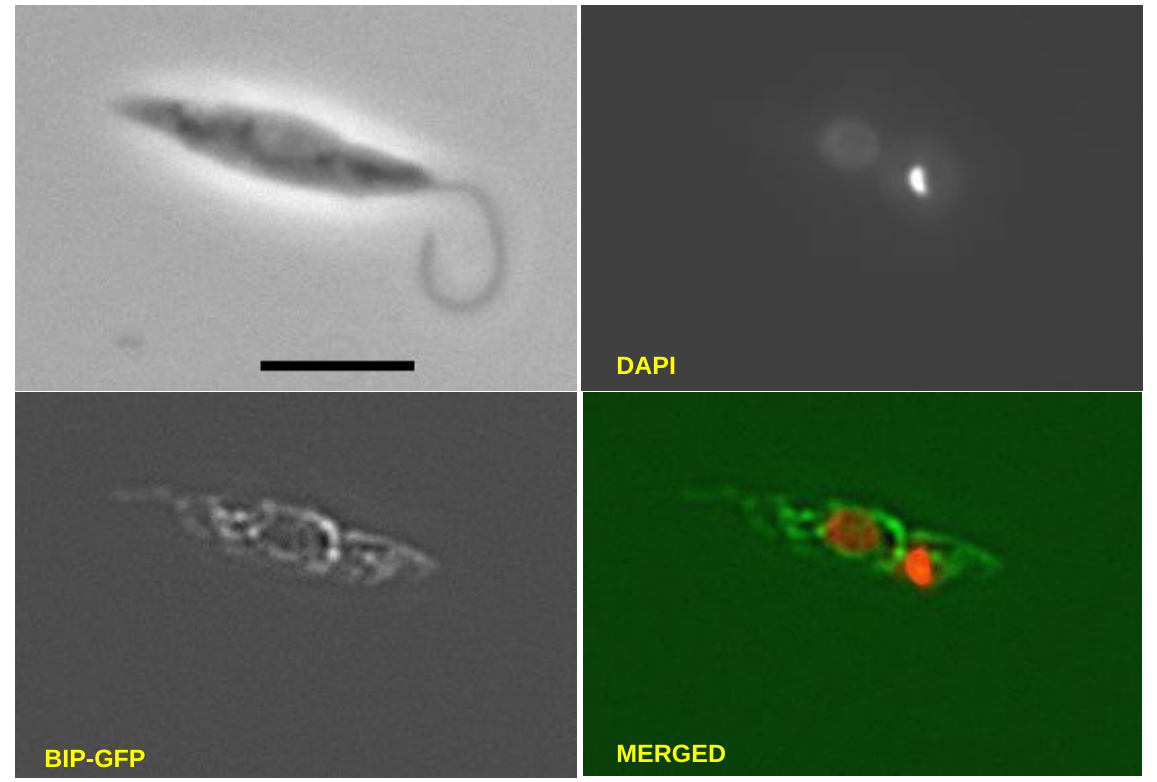

DAPI
MERGED
BIP-GFP

Supplement: Additional File 2 — Visualisation of the endoplasmic reticulum (ER) in Leishmania major using an ER-marker. The pictures provided show an L. major cell where the ER was visualised using an ER-specific expression plasmid construct. Images of an L. major promastigote form expressing the plasmid construct GFP-MDDL that acts as an endoplasmic reticulum retention signal in trypanosomatids [31]. Upper left: phase contrast microscopy; scale bar : 10 microns. Upper right (DAPI): DAPI-staining of the nucleus and kinetoplast. Lower left (BIP-GFP): Localisation of the GFP-MDDL marker viewed in fluorescence. Lower right (MERGE): Colour combination of GFP (green) and DAPI (red) fluorescence. [file 1471-2164-8-57-S2.ppt]
